# Comparative Performance of the C-reactive Protein-to-Albumin Ratio (CAR) and the Thrombolysis in Myocardial Infarction (TIMI) Score in Predicting Major Adverse Cardiac Events (MACE) Among Patients With ST-Elevation Myocardial Infarction (STEMI)

**DOI:** 10.7759/cureus.91175

**Published:** 2025-08-28

**Authors:** Madhu Mathi, Srinivasan Ramadurai, Rajkumar Mani, Bhargavi Mathan, Suresh kumar R.

**Affiliations:** 1 General Medicine, Sri Ramachandra Institute of Higher Education and Research, Chennai, IND; 2 Internal Medicine, Sri Ramachandra Medical College, Sri Ramachandra Institute of Higher Education and Research, Chennai, IND; 3 Internal Medicine, Sri Ramachandra Institute of Higher Education and Research, Chennai, IND

**Keywords:** : acute coronary syndrome, c-reactive protein to albumin ratio, major adverse cardiac events (mace), st-elevation myocardial infarction (stemi), thrombolysis in myocardial infarction

## Abstract

Background

Acute coronary syndrome (ACS) is a common emergency presentation. The C-reactive protein-to-albumin ratio (CAR) serves as a composite marker reflecting both systemic inflammation and nutritional status and may enhance prognostic accuracy in myocardial infarction (MI).

Objective

To assess the prognostic utility of the CAR in predicting disease severity and major adverse cardiac events (MACE) among patients with ST-elevation myocardial infarction (STEMI).

Methodology

A cross-sectional, observational, prospective study was conducted on 203 patients with STEMI who presented to the outpatient and emergency departments between November 2023 and May 2025. Demographic and clinical data were collected. Thrombolysis in myocardial infarction (TIMI) scores were calculated, and serum C-reactive protein and albumin levels were measured within 24 hours of admission. The primary outcome was in-hospital mortality, while MACE, including reinfarction, revascularization, heart failure, and death, was assessed at 28 days through follow-up.

Results

Among the participants, the in-hospital mortality rate was 14 (6.9%), and the 28-day incidence of MACE was 38 (18.7%). Notably, MACE occurred more frequently in patients with elevated CAR, even among those with lower TIMI scores, suggesting that CAR may be a more sensitive early predictor of adverse outcomes. CAR demonstrated superior prognostic performance, with an area under the curve (AUC) of 0.726 compared with 0.668 for the TIMI score.

Conclusions

A CAR threshold greater than 0.568 was identified as clinically useful for early risk stratification in patients with STEMI. These findings support the integration of CAR into routine assessments to improve early prognostic evaluation and guide clinical decision-making.

## Introduction

Acute coronary syndrome (ACS) is a frequent clinical emergency encompassing ST-elevation myocardial infarction (STEMI), non-ST-elevation myocardial infarction (NSTEMI), and unstable angina, differentiated by electrocardiogram (ECG) changes and cardiac biomarkers [1]. STEMI, caused by total coronary artery occlusion, leads to irreversible myocardial injury if not rapidly treated. The global burden of myocardial infarction (MI)-related mortality remains high, influenced by comorbidities such as diabetes, hypertension, dyslipidemia, and lifestyle habits like smoking and alcohol use [2]. Effective prognostication is vital for optimizing treatment and reducing mortality. Conventional biomarkers, such as cardiac troponins, creatine kinase-myocardial band (CK-MB), C-reactive protein (CRP), N-terminal pro-B-type natriuretic peptide (NT-proBNP), WBC count, and D-dimer, are routinely used to assess the severity and predict complications of MI [3].

CRP is an acute-phase reactant synthesized by the liver in response to systemic inflammation and has been strongly associated with adverse outcomes after MI, including reinfarction and heart failure [2,3]. Serum albumin, a negative acute-phase reactant, reflects nutritional and inflammatory status and is inversely correlated with cardiovascular risk. Hypoalbuminemia after MI is linked to increased mortality and complications, particularly heart failure [4]. The CRP-to-albumin ratio (CAR) combines the predictive capabilities of both markers, offering insight into both inflammation and general health. CAR has been increasingly recognized as a powerful prognostic indicator in cardiovascular diseases, especially in patients with ACS and STEMI [1].

Traditional risk stratification tools, such as the thrombolysis in myocardial infarction (TIMI) score, guide management decisions in patients with STEMI by incorporating clinical and hemodynamic variables [2]. However, these scores may overlook the dynamic inflammatory and metabolic status of patients. CAR, by reflecting both systemic inflammation and nutritional reserve, may provide more value in risk assessment. Studies by Celik et al. [5] and Liu et al. [6] have shown that CAR better predicts adverse outcomes, such as heart failure and reinfarction, than TIMI, particularly in patients with an elevated inflammatory state [1]. This study aims to compare CAR and the TIMI score in predicting major adverse cardiac events (MACE) in STEMI, potentially enhancing early risk stratification and individualized care.

The objective of this study was to evaluate the prognostic utility of the CAR in predicting disease severity and MACE among patients with STEMI.

## Materials and methods

Study settings and design

A cross-sectional, observational, and prospective study was conducted among patients presenting to the Emergency Room (ER) and Outpatient Department (OPD) of Sri Ramachandra Medical College (SRMC), a tertiary care hospital. The study period spanned from August 1, 2023, to May 31, 2025.

Sample size and sampling method

The sample size was calculated using the formula:

\[ n = \frac{\text{DEFF} \cdot Np(1-p)}{\left(\frac{d^{2}}{Z^{2}_{1-\alpha/2}}(N-1) + p(1-p)\right)} \]

where the population size (*N*) was 300 (based on Medical Records Department (MRD) data from 2020 to 2023 at Sri Ramachandra Institute of Higher Education and Research (SRIHER), representing the total number of patients who underwent PCI during this period). The hypothesized frequency of the outcome factor (mortality, based on previous MRD records) was *p* = 5.79%. The confidence limits (d) were set at 5%, and the design effect (DEFF) was 1. Using this formula, the calculated sample size was 68.However, a total of 203 patients who met the inclusion criteria during the study period were included in the analysis.

Participants selection

Patients who presented to the ER or OPD at SRMC with chest pain or angina-equivalent symptoms and were diagnosed with STEMI based on ECG and laboratory findings (Troponin, B-type natriuretic peptide (BNP), creatine kinase-myocardial band (CK-MB)) were included in the study. Demographic data and baseline clinical history were obtained from patient records, and the TIMI risk score was calculated for each individual. Blood samples were collected within 24 hours of hospitalization to measure CRP. Albumin values were obtained from the patient’s routine liver function tests. Based on these values, CAR was calculated within the first 24 hours of hospitalization.

Inclusion criteria

Patients who presented with chest pain or angina-equivalent symptoms to the ER or OPD (Cardiology and Medicine) and were diagnosed with STEMI based on electrocardiographic findings.

Exclusion criteria

Patients with the following conditions were excluded from the study: known chronic pancreatitis, malignancy, chronic liver disease, other acute or chronic inflammatory disorders, hematological disorders, or acute infections. Patients with incomplete data and those with a body mass index (BMI) <18.5 were also excluded.

Laboratory measurements

Blood was collected in red colored vacutainer and shifted to the laboratory in a cold box within 20 minutes of sample collection. Stored samples (>30 minutes) were discarded. Routine complete blood cell count and blood chemistry measurements, including albumin (g/L) and CRP (mg/L), were performed on a blood sample obtained on admission. Cardiac troponin I (ng/L) and CK-MB (U/L) levels were measured using a point-of-care card test. The CRP levels were measured using a spectrophotometer or an automated immunoassay. 

Outcome

The primary outcome of this study was in-hospital mortality. Secondary outcomes included reinfarction, revascularization, and heart failure. All in-hospital events were determined from hospital files and electronic medical records at the time of discharge. For the evaluation of secondary outcomes, follow-up was performed through direct interviews, telephone contacts, outpatient clinic visits, and/or review of the hospital electronic database after 28 days of the STEMI episode. The primary outcome of the study was major adverse cardiac events (MACE), which included reinfarction, revascularization, heart failure, and mortality. Outcome prediction was compared using the TIMI risk score and the CAR.

Definitions

STEMI was defined based on the following criteria: (1) ongoing ischemic symptoms within 12 hours, (2) a typical rise or fall in cardiac biomarkers, and (3) new ST elevation in at least two contiguous leads, with 0.2 mV in leads V1, V2, and V3 or 0.1 mV in the remaining leads, or a newly developed left bundle branch block pattern [7].

Myocardial reinfarction was defined as the recurrence of typical clinical symptoms and new electrocardiographic changes, with either a new elevation of CK-MB levels to more than twice the upper normal limit or an increase of at least 50% from a previously elevated level, occurring within 28 days of an incident MI. If an MI occurred more than 28 days after an incident MI, it was termed a recurrent MI [8]. Revascularization was defined as repeat PCI or bypass grafting involving not only the infarct-related artery but also non-infarct-related arteries, driven by ischemic symptoms. New-onset heart failure was defined as the occurrence of heart failure with symptoms consistent with New York Heart Association (NYHA) class III-IV, developing within 24 hours after the index event.

Heart Failure

Heart failure is a clinical syndrome with symptoms and/or signs caused by a structural and/or functional cardiac abnormality, resulting in reduced cardiac output and/or elevated intracardiac pressures at rest or during stress [9].

Tools for data collection

A predesigned and pretested semi-structured questionnaire was used to determine the baseline and clinical profile of study participants.

Pretesting of the Semi-structured Questionnaire

The semi-structured questionnaire and scoring system were pretested on 10% of the total sample size. Necessary modifications were made to the schedule, and data collection was then carried out.

Ethical consideration

Institutional ethical clearance was obtained before the commencement of data collection. Informed consent was obtained from the patients. Data collected from patients were kept confidential. The institutional ethical clearance number was CSP-MED/23/JUL/89/154.

Statistical analysis

Data were analyzed using SPSS software, version 23 (IBM Corp., Armonk, NY). The categorical variables were represented in the form of frequency tables. Mean and standard deviation were used as measures of central tendency for continuous variables. Tests of significance, such as the chi-square test, were used to determine differences in frequency distributions between two or more groups of a given independent variable. Binary logistic regression analysis was conducted to determine independent predictors of MACE. The TIMI risk score for STEMI is a simple additive bedside score (0-14 points) based on 10 baseline variables: age 65-74 years (2 points) or ≥75 years (3 points); systolic blood pressure <100 mmHg (3 points); heart rate >100 bpm (2 points); Killip class II-IV (2 points); weight <67 kg (1 point); history of diabetes, hypertension, or angina (1 point); anterior ST elevation or left bundle branch block (1 point); and time to treatment >4 hours (1 point). Higher total scores indicate a graded and substantially increased risk of mortality and adverse outcomes. The TIMI score is intended for rapid risk stratification at presentation to guide early management. Based on the study by Antman et al. [10], the TIMI score was categorized as mild (0-2), moderate (3-4), and severe (≥5). Receiver operating characteristic (ROC) curve analysis was applied to evaluate the predictive value of CAR for MACE. Using Youden’s index, which identifies the point on the ROC curve that maximizes the sum of sensitivity and specificity, the optimal cut-off values for CAR were determined. This method yielded three distinct risk categories: low risk (CAR ≤ 0.50), intermediate risk (CAR 0.51-7.54), and high risk (CAR > 7.55). These thresholds represent clinically relevant breakpoints where the likelihood of MACE significantly changes, enabling stratification of patients into meaningful prognostic groups.

## Results

Table 1 presents the baseline characteristics of the study participants. The majority were aged between 40-58 years (91, 44.8%) and 59-78 years (90, 44.3%), while 13 (6.4%) were 39 years or younger and 9 (4.4%) were 79 years or older. The mean age was 56.68 ± 10.86 years, with a 95% confidence interval ranging from 45.82 to 67.54 years. Among the 203 participants, 136 (67.0%) were male and 67 (33.0%) were female, indicating a male predominance. Most patients (160, 78.8%) presented with chest pain, while 43 (21.2%) reported shortness of breath. Diabetes mellitus was the most common comorbidity, affecting 103 (50.7%) of the population, with an almost equal split between diabetic and non-diabetic individuals. Systemic hypertension was observed in 96 (47.3%) participants, and dyslipidemia was present in 61 (30%), making it less prevalent than diabetes or hypertension. As shown in Table 1, the most common BMI category was normal weight (67, 33.0%), followed closely by overweight (63, 31.0%). Obesity (including Obesity Classes I and II) was observed in 27.1%, reflecting a substantial proportion at heightened cardiometabolic risk, while only 8.9% were underweight, indicating a relatively low burden of undernutrition.

**Table 1:** Distribution of study participants according to their baseline profiles (n = 203). BMI, body mass index

Characteristics	Sub-categories	*n* (%)
Age (in completed years) (*n* = 203)	<=39 years	13 (6.4%)
	40-58 years	91 (44.8%)
	59-78 years	90 (44.3%)
	>79 years	9 (4.4%)
Mean age of diagnosis (in years)	56.68 ± 10.86	
Gender of the study participants	Male	136 (67.0%)
	Female	67 (33.0%)
Substance abuse (*n* = 96)	Alcohol	45 (22.17%)
	Smoking	51 (25.12%)
Time to initiate treatment	<4 hours	143 (70.4%)
	>4 hours	60 (29.6%)
Chief complaints	Chest pain	160 (78.8%)
	SOB	43 (21.2%)
Comorbidities	Diabetes mellitus	103 (50.7%)
	Systemic hypertension	96 (47.3%)
	Dyslipidemia	61 (30%)
BMI	Underweight (<18.5 kg/m^2^)	18 (8.9%)
	Normal (18.5-22.9 kg/m^2^)	67 (33.0%)
	Overweight (23-24.9 kg/m^2^)	63 (31.0%)
	Obesity I (25-29.9 kg/m^2^)	40 (19.7%)
	Obesity II (>30 kg/m^2^)	15 (7.4%)

Table 2 shows the biochemical parameters of the study participants. The mean hemoglobin level was 12.43 g/dL (range: 8.0-16.40 g/dL), while the total white blood cell count varied widely, with a mean of 10,928 ± 3,703 cells/mm³ (range: 4,879-19,600 cells/mm³). Platelet count was 2.88 ± 0.90 × 10^5 cells/mm³ (1.10-5.87 × 10^5 cells/mm³). Renal function markers included blood urea nitrogen, which averaged 12.58 ± 4.22 mg/dL (1.28-27.0 mg/dL), and serum creatinine, which averaged 1.13 ± 0.54 mg/dL (0.40-1.80 mg/dL). Inflammatory status, as measured by C-reactive protein, exhibited the greatest variability (mean 5.76 ± 9.18 mg/L; range 0.20-45.40 mg/L), whereas serum albumin levels were relatively stable at 3.74 ± 0.58 g/dL (range 2.10-4.80 g/dL), reflecting the overall nutritional status of the study participants.

**Table 2:** Biochemical parameters of study participants. BUN, blood urea nitrogen

Biochemical parameters	Mean ± SD	95% confidence interval	
		Minimum-maximum	Reference range
Hemoglobin	12.43 ± 2. 26 g/dL	8.0-16.40	12-17 g/dL
Total count	10,928 ± 3,703/cmm	4,879-19,600	4,000-11,000/cmm
Platelet	2.88 ± 0.90/cmm	1.10-5.87	1.5-4.5 lakhs/cmm
BUN	12.58 ± 4. 22 mg/dL	1.28-27.0	7-18 mg/dL
Creatinine	1.13 ± 0.54 mg/dL	0.40-1.8	0.6-1.3 mg/dL
C-reactive protein	5.76 ± 9.18 mg/L	0.20-45.40	0-5 mg/L
Albumin	3.74 ± 0.58 gm/dL	2.10-4.80	3.2-4.8 gm/dL

Table 3 shows that in this study, 189 (93.1%) patients were in stable condition, whereas 14 (6.9%) had died at the time of discharge. Among the causes of in-hospital mortality, cardiogenic shock was the most common, accounting for 10 cases (71.4%), followed by reinfarction in three cases (21.4%) and heart failure in one case (7.2%). The frequency and percentage distribution of MACE observed in the study population at 28 days follow-up showed an overall mortality rate of 39.4%, making death the most frequent MACE event in the cohort. Reinfarction (9, 23.7%) and heart failure (8, 21.1%) were relatively common complications. Cardiogenic shock was the least frequent MACE event (6 cases, 15.8%) but remained a critical adverse outcome.

**Table 3:** Outcome of study participants at the time of discharge and 28 days follow-up.

	Risk factors	Frequency (*n*)	Percentage (%)
Status at discharge	Stable	189	93.1
	Mortality	14	6.9
Causes of mortality during discharge (*n *= 14)	Reinfarction	3	21.4
	Cardiogenic shock	10	71.4
	Heart failure	1	7.2
MACE at 28 days of follow-up (*n *= 38)	Reinfarction	9	23.7
	Cardiogenic shock	6	15.8
	Heart failure	8	21.1
	Death	15	39.4

Tables 4-5 show that chi-square analysis revealed a significant association between CAR and MACE at 28-day follow-up in patients with STEMI (χ² = 38.479, *P* < 0.001). Patients in the mild CAR group (≤0.50) had low rates of reinfarction (1.8%), revascularization (4.5%), heart failure (0.9%), and death (0.9%). In contrast, the moderate CAR group (0.51-7.54) showed higher incidences of reinfarction (8.0%), heart failure (8.0%), and death (13.8%), with no revascularization. The severe CAR group (>7.55) had the highest mortality rate (33.3%) and a 16.7% revascularization rate, but no reinfarction or heart failure.

**Table 4:** Association between CAR severity and MACE outcome at 28 days follow-up. Percentages are calculated within each risk category. CAR, C-reactive protein-to-albumin ratio; MACE, major adverse cardiac events

CAR risk score (Based on ROC cut points)	Reinfarction, *n *(%)	Revascularization, *n *(%)	Heart failure, *n *(%)	Death, *n *(%)	Stable, *n *(%)	Total (*n*)	*X*^2^	*P*-value
Mild (≤0.5)	2 (1.8%)	5 (4.5%)	1 (0.9%)	1 (0.9%)	101 (91.9%)	110	38.479	<0.001
Moderate (0.51-7.54)	7 (8.0%)	0 (0.0%)	7 (8.0%)	12 (13.8%)	61 (70.2%)	87		
Severe (>7.55)	0 (0.0%)	1 (16.7%)	0 (0.0%)	2 (33.3%)	3 (50.0%)	6		
Total	9	6	8	15	165	203		

**Table 5:** Association between TIMI severity and MACE outcome at 28 days follow-up. Percentages are calculated within each risk category. MACE, major adverse cardiac events; TIMI, thrombolysis in myocardial infarction

TIMI risk	Reinfarction, *n* (%)	Revascularization, *n* (%)	Heart failure, *n* (%)	Death, *n* (%)	Stable, *n* (%)	Total (*n*)	*X*^2^	*P* -value
Mild (0-2)	0 (0.0%)	0 (0.0%)	1 (5.3%)	3 (15.8%)	15 (78.9%)	19	12.54	0.128
Moderate (3.1-4.9)	4 (3.5%)	1 (0.9%)	3 (2.7%)	9 (8.0%)	96 (84.9%)	113		
Severe (≥5)	5 (7.0%)	5 (7.0%)	4 (5.7%)	3 (4.2%)	54 (76.1%)	71		
Total	9 (4.4%)	6 (3.0%)	8 (3.9%)	15 (7.4%)	165 (81.3%)	203		

In comparison, the TIMI risk score was not significantly associated with MACE (χ² = 12.54, *P* = 0.128), although there was a trend toward increasing events with higher TIMI scores, particularly reinfarction (55.6%), revascularization (83.3%), and heart failure (50.0%) in the severe category. Notably, higher mortality was observed in the moderate TIMI group (60.0%).

These findings suggest that CAR has a significant association with predicting MACE at 28-day follow-up, compared to the TIMI score.

**Figure 1:**
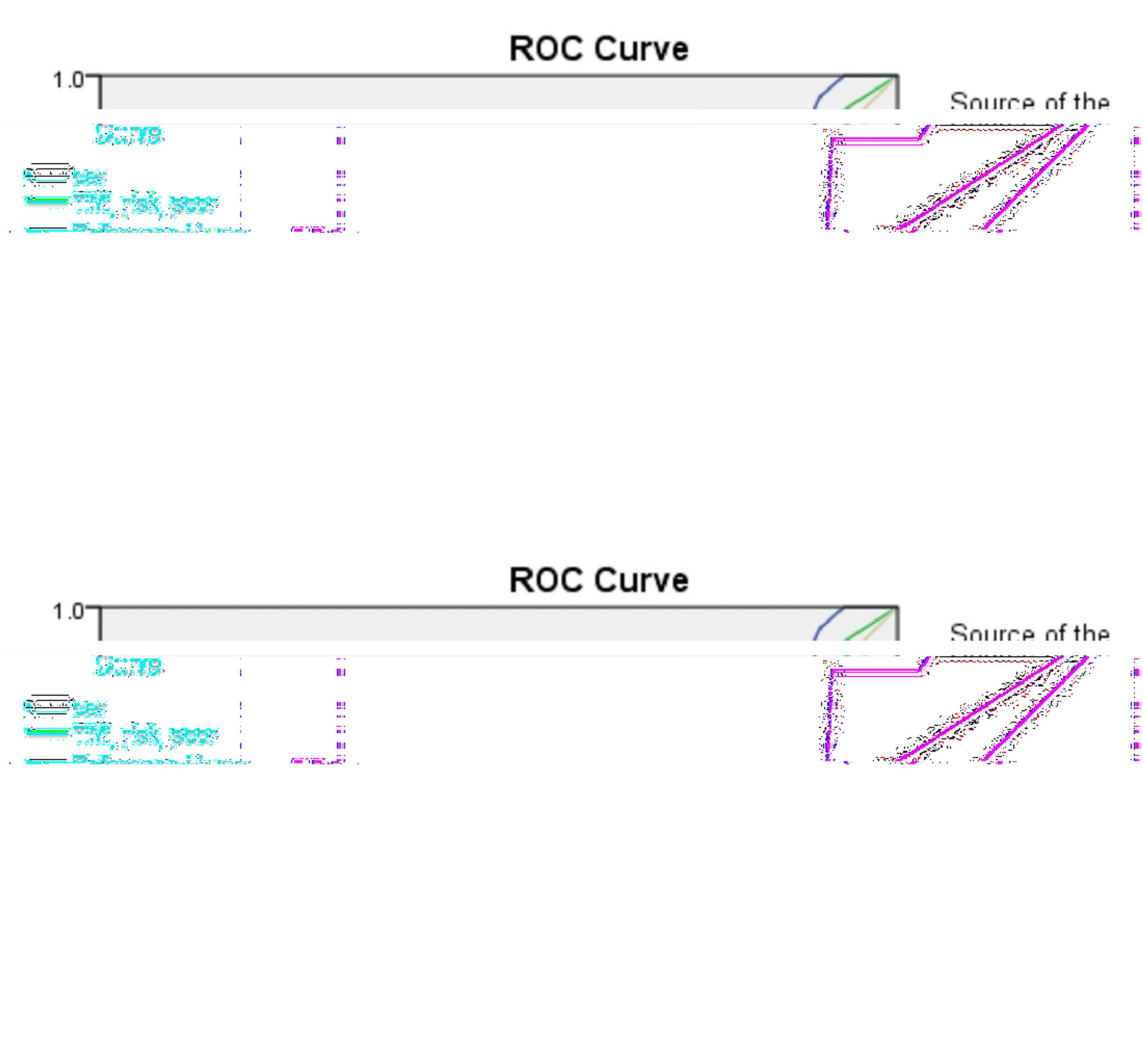
Comparison of CAR and TIMI score using the receiver operating characteristic curve in predicting MACE. MACE, major adverse cardiac events; TIMI, thrombolysis in myocardial infarction; CAR, C-reactive protein-to-albumin ratio

Figure 1 shows that ROC analysis demonstrated that CAR had a fair ability to predict 28-day MACE in patients with STEMI, as indicated by an AUC of 0.726. The best-performing cutoff value identified was CAR > 0.568, which yielded a sensitivity of 76.3% and a specificity of 63.6%. This threshold corresponded to the highest Youden’s index and serves as a practical point for clinical risk assessment. Patients exceeding this CAR level were notably more prone to complications such as reinfarction, heart failure, the need for revascularization, or death. By contrast, the TIMI risk score had a lower AUC of 0.668, indicating a somewhat reduced predictive capability, as shown in Table 6. Although both CAR and TIMI scores are useful for assessing patient risk, CAR appeared to perform better, particularly in identifying individuals at higher risk who may benefit from early, targeted management strategies.

**Table 6:** AUC for CAR and TIMI risk category in predicting MACE. MACE, major adverse cardiac events; TIMI, thrombolysis in myocardial infarction; CAR, C-reactive protein-to-albumin ratio; AUC, area under the curve; CI, confidence interval

Scores	AUC	Standard error	Asymptotic 95% CI	
			*P*-value	95% CI
CAR	0.726	0.046	<0.001	0.636-0.816
TIMI	0.668	0.053	0.001	0.563-0.773

Table 7 shows that binary logistic regression analysis identified CAR category, time to treatment, and hypertension as significant predictors of MACE outcomes. CAR demonstrated a strong inverse association with adverse events, with patients in the mild CAR group having 81% lower odds of experiencing MACE compared to those in the moderate or severe categories (odds ratio (OR) = 0.192, *P* = 0.009).

**Table 7:** Regression analysis showing predictors of MACE in patients with STEMI. MACE, major adverse cardiac events; STEMI, ST-elevation myocardial infarction; TIMI, thrombolysis in myocardial infarction

Factors	Coefficient	SE	*P*-valve	95% CI	
				Upper	Lower
TIMI risk category	4.126	0.790	0.73	0.877	19.416
CAR category	0.410	0.481	0.009	0.055	0.668
Time to treatment - Early	0.199	0.470	0.001	0.079	0.501
Hypertension - no	0.398	0.454	0.042	0.163	0.969
CAD	0.590	0.516	0.307	0.215	1.622
Diabetes mellitus	0.641	0.487	0.361	0.247	1.665
Dyslipidemia	2.280	0.505	0.103	0.848	6.130
Substance use	1.177	0.546	0.765	0.404	3.429

Delayed treatment was associated with a significantly increased risk of MACE, whereas early intervention reduced the odds by 81% (OR = 0.199, *P* = 0.001). Interestingly, hypertension appeared to be a protective factor; patients without hypertension had 60.2% lower odds of adverse outcomes (OR = 0.398, *P* = 0.042).

Although the TIMI risk category was statistically significant overall (*P* = 0.010), the increased odds observed in the mild group (OR = 4.126, *P* = 0.073) did not reach individual significance.

These findings underscore the importance of inflammatory markers, timely intervention, and comprehensive clinical profiling in predicting 28-day outcomes in patients with STEMI.

## Discussion

This study evaluated the predictive value of CAR compared to the TIMI score in the prediction of MACE in patients with STEMI. The study cohort comprised 203 patients with STEMI from SRIHER. The majority (91, 44.8%) were aged between 40 and 58 years, with a mean age of 56.7 years - a distribution closely mirroring findings from the CREATE registry by Xavier et al., which reported a mean age of approximately 57.5 ± 12.1 years in Indian patients with ACS. Males predominated (136, 67.0%), consistent with the marked male predominance reported by Xavier et al. [11].

Among the 203 patients enrolled, in-hospital mortality was 6.9%, and 18.3% experienced MACE within 28 days. CAR showed improved predictive accuracy over the TIMI score, with a higher area under the ROC curve (AUC: 0.726 vs. 0.668). Thus, CAR is as good a biomarker for early risk stratification and MACE prediction as the TIMI score. Increased CAR values were strongly related to higher rates of reinfarction, heart failure, and mortality. These conclusions are consistent with findings by Acet et al. and Çınaret al. [12,13]. Conversely, while the TIMI score continues to be a popular practical tool [10,14], its inability to incorporate inflammatory markers could potentially limit its ability to capture the complete pathophysiological risk spectrum.

Hypertension, late treatment, and elevated CAR were significant predictors of adverse outcomes according to binary logistic regression [15]. Early treatment lowered the risk of MACE substantially, highlighting the importance of early reperfusion. Non-hypertensive individuals had fewer complications, indicating the complex interplay between chronic diseases and outcomes in STEMI. These results align with earlier studies linking inflammation to myocardial damage and plaque instability [2].

Timely intervention is critical for reducing STEMI-associated morbidity and mortality. The study found that 70.4% of patients received treatment within four hours of symptom onset, while 29.6% experienced delays. The primary reasons for delayed treatment included low perceived symptom severity (12.8%), geographical barriers (10.8%), and logistical issues (5.9%). Similar findings were reported in the Kerala STEMI Registry [16], which highlighted that only 10% of eligible patients received timely reperfusion therapy. These findings underscore the importance of patient education on symptom recognition, healthcare accessibility improvements, and streamlined emergency response systems to mitigate delays in treatment initiation. Addressing these factors could improve early intervention rates and enhance patient outcomes.

CAR is easy to use, affordable, and directly related to patient outcomes. It could serve as a valuable supplement to current risk scores. Incorporating CAR into early risk assessments may help clinicians more precisely identify high-risk patients and expedite treatment decisions.

Patients with high CAR levels tended to have increased rates of MACE, including in-hospital mortality, arrhythmias, cardiogenic shock, and reinfarction. This is consistent with prior studies demonstrating CAR’s usefulness in ACS risk assessment [17,18]. Kundi et al. [18] found that CAR outperformed CRP or albumin alone as an independent and robust long-term mortality predictor. Similarly, Zhou et al. showed that elevated CAR levels were associated with higher in-hospital MACE rates after STEMI [17].

While well established and in common use, the TIMI score relies on clinical factors such as age, blood pressure, history of risk, and ECG findings [14]. Although it remains valuable, it may not fully capture metabolic and inflammatory changes influencing STEMI outcomes. In comparison, CAR demonstrated a similar or greater predictive value in our study.

CAR also offers practical advantages. It requires only routine blood tests and no patient input, making it feasible even in urgent situations.

Limitations 

This study has several important limitations. First, the relatively small sample size (*n *= 203) may limit statistical power and reduce the reliability and generalizability of the findings to the broader population. Although the cohort was carefully defined, it may not fully represent the general public, introducing the possibility of selection bias. Second, while we have now expanded and clarified the methodological details, including explicit definitions of outcome measures and exclusion criteria, these factors still restrict external validity and should be acknowledged as potential sources of bias. Third, although existing literature supports the utility of CAR as a prognostic biomarker in patients with STEMI, most published studies are retrospective in nature; hence, larger, multicenter, prospective trials are required to validate and extend these findings. Finally, although CAR is simple to calculate, its practical application in acute care may be limited by the availability of rapid CRP and albumin assays in certain settings. Taken together, these limitations suggest that the present findings should be interpreted with caution, while still providing important preliminary insights.

## Conclusions

The study found that while the usefulness of the TIMI scoring system may not be questioned, very rigorous risk stratification will play a vital role in patients with STEMI. On the other hand, CAR exhibited a greater value in the prediction of MACE. Thus, timely interventions, targeted risk mitigations, and inflammation-based assessments must be prioritized in patient management flow to improve outcomes. Our study supports the previous investigations regarding the utility of CAR in cardiovascular risk assessment and recommends incorporating it in clinical decision-making pathways.
